# Hemolysis and acquired methemoglobinemia associated with lidocaine and benzocaine topical application: a case report

**DOI:** 10.1186/s13256-023-03898-x

**Published:** 2023-04-20

**Authors:** Nasim Khajavirad, Ghazal Daftari, Mehrasa Raisi Jelodar

**Affiliations:** 1 Department of Internal Medicine, Imam Khomeini Hospital Complex, Tehran University of Medical Science, Tehran, Iran; 2 School of Medicine, Tehran University of Medical Science, Keshavarz Boulevard, Tehran, 1419733141 Iran; 3 ͨBabol University of Medical Science, Babol, Iran

**Keywords:** Topical anesthetics, Methemoglobin, Lidocaine, Benzocaine, Hemolysis

## Abstract

**Background:**

Topical anesthetics are commonly used over the counter, and one of the adverse effects of these medications is methemoglobinemia, which is a serious and life-threatening condition.

**Case presentation:**

We describe a 25-year-old Persian male presenting with generalized weakness, dizziness, headache, and cyanosis. In addition, he had genital warts starting 3 weeks ago, which were self-treated with podophyllin, resulting in itching and pain. He used over-the-counter topical anesthetics, including benzocaine and lidocaine, to reduce the symptoms. According to the lab data, signs and symptoms of methemoglobinemia and hemolysis were diagnosed. Considering the hemolysis, ascorbic acid was used for treatment. The patient was discharged after 5 days with normal arterial blood gas and pulse oximetry and no signs and symptoms.

**Conclusion:**

This case highlights that self-administration of some topical anesthetics may lead to potentially fatal conditions.

## Introduction

Hemoglobin A (Hb), the main type of the hemoglobin in adolescents, contains ferrous iron (Fe^2+^) and four heme groups [1]. Oxidization of the ferrous iron (Fe^2+^) to ferric iron (Fe^3+^) produces methemoglobin [1]. Methemoglobin is incapable of binding, transporting, and releasing oxygen into the tissues effectively, resulting in hypoxia and functional anemia [2]. Physiological mechanisms and enzymatic systems protect the red blood cells against oxidative stress and try not to let the methemoglobin levels exceed 1–2% of the total hemoglobin [2, 3]. Methemoglobinemia, which means high levels of methemoglobin in the blood, is a rare but life-threatening condition, presenting in hereditary or acquired forms [4]. Hemoglobin M disease, cytochrome b_5_ reductase deficiency, and glucose-6-phosphate dehydrogenase (G6PD) deficiency are causes of hereditary methemoglobinemia [5]. Furthermore, some drugs, such as dapson, nitroglycerine, nitroprusside, nitric oxide, sulfanamides, phenazopyridine, chloroquine, lidocaine, prilocaine, and benzocaine, may cause acquired methemoglobinemia [4, 5]. Cocaine-derived anesthetics, including lidocaine and benzocaine, are extensively used in medical procedures, such as endoscopy and bronchoscopy [6]; moreover, they may be used topically [6]. In this study, we present a unique and atypical case of methemoglobinemia and hemolysis induced by self-administration of topical lidocaine and benzocaine.

## Case presentation

A 25-year-old self-employed Persian male with no past medical history presented to the hospital with generalized weakness, dizziness, and headache for almost 7 days. After an accurate history taking, he also reported some genital warts that appeared starting 3 weeks ago. He started self-treatment of the warts with podophyllin, which is an over-the-counter topical ointment. Self-administration of the excessive podophyllin caused severe pain and itching of the genitalia. To reduce the noticed symptoms, he started using over-the-counter topical anesthetics, such as benzocaine and lidocaine, for approximately 2 weeks. The patients surprisingly reported that he used four ointment tubes daily: two tubes of benzocaine ointment 5% (60 g/10 g) and two tubes of lidocaine ointment 5% (70 g/10 g). He did not smoke, and his family history of polycythemia and hematologic, cardiovascular, and pulmonary disorders was negative. On physical examination, he was alert and not distressed, and skin pallor and perioral and acral cyanosis were remarkable. Examination of the heart and lungs was normal. The vital signs revealed 37 °C (98.6 °F), blood pressure of 115/75 mmHg, heart rate of 80 beats per minute, and respiration of 18 breaths per minute. Oxygen saturation (SpO_2_) was 75% on room air, increasing to almost 85% when using an oxygen mask. Electrocardiogram (ECG) and chest X-ray were normal. Laboratory findings indicated that anemia and renal and liver functional tests and inflammatory markers [erythrocyte sedimentation rate (ESR) and C-reactive protein (CRP)] were normal. Additionally, the urine analysis was not remarkable, and the urine culture was negative. G6PD was sufficient. Viral markers, including human immunodeficiency virus (HIV) Ab, hepatitis C virus (HCV) Ab, hepatitis B surface (HBS) Ag, and HBS Ab were nonreactive. Prothrombin time (PT), activated partial thromboplastin time (APTT), and international normalized ratio (INR) were normal. Furthermore, the lactate dehydrogenase (LDH) and the total and direct bilirubin increased up to 3500 U/L, 2.8 mg/dL, and 1.2 mg/dL, respectively. The arterial blood gas (ABG) had chocolate-brown appearance and measured pH of 7.51, pCO_2_ 26.8 mmHg, pressure of O_2_ (pO_2_) of 189.2 mmHg, HCO_3_^−^ of 21.1 mEq/L, and arterial oxygen saturation (SaO_2_) of 99.4%. CO-oximetry revealed total calculated hemoglobin of 7.5 g/dL, oxyhemoglobin of 70.9%, carboxyhemoglobin of 8.2%, and methemoglobin of 19.5%. Considering that abnormal LDH and bilirubin levels may be due to hemolysis, direct and indirect Coombs tests were taken, which were negative, and splenomegaly was not detected in abdominopelvic ultrasonography. Considering the normal cardiovascular and pulmonary findings (ECG and chest X-ray) and according to the excessive application and the discrepancy between SaO_2_ and SpO_2_, the final diagnosis was lidocaine- and benzocaine-induced methemoglobinemia and hemolysis. According to the hemolysis, methylene blue was not the choice of the treatment. The anesthetic ointment was discontinued, and 3 g ascorbic acid (vitamin C) was intravenously administered daily for 3 days. After 2 days, repeated CO-oximetry showed decreased methemoglobin (12.3%) and increased oxyhemoglobin (76.1%). The cyanosis and hemolysis symptoms disappeared within 3 and 5 days, respectively. At the time of discharge, all signs and symptoms were resolved so that SpO_2_ was 97–98%, LDH and bilirubin levels returned to normal ranges, methemoglobin was 0.3%, and oxyhemoglobin increased up to 91.6%.

## Discussion

The acquired form of methemoglobinemia may be induced by medication overdose following ingestion, skin absorption, and inhalation [4]. Drugs may oxidize the hemoglobin directly or indirectly by producing superoxide free radicals [4]. Route of administration, prolonged use, body surface area, age, accompanying use of oxidative drugs, comorbidity, and high doses of oxidizing drugs are related to the occurrence and severity of methemoglobinemia [4]. A methemoglobin level up to 15% of total hemoglobin is asymptomatic in healthy patients; headache and fatigue is associated with a level ranging from 15% to 30%; dizziness, dyspnea, and syncope occur at levels of 30–50%; and at levels higher than 50%, central nervous system (CNS) depression, coma, and death are expected [2, 7]. Symptoms may be worsened due to cardiovascular, hematologic, and pulmonary disorders, as well as infection and renal or liver failure [4]. Prilocaine, lidocaine, and benzocaine are anesthetics that may cause methemoglobinemia [8]. In the current case, the excessive use of topical benzocaine and lidocaine resulted in methemoglobinemia presenting with dizziness, cyanosis, and generalized weakness. Nappe *et al.* reported a case of methemoglobinemia in a patient who used benzocaine gel three times daily for 3 days [7]. The gel was self-administered to reduce the toothache [7]. One study conducted by Hahn *et al.* demonstrated that the use of five tubes of EMLA cream (lidocaine and prilocaine) before laser epilation resulted in methemoglobinemia [9]. Furthermore, Lavergne *et al.* [10] presented a case of methemoglobinemia and acute hemolysis induced by tetracaine lozenges. Several studies reported methemoglobinemia induced by inhalation of anesthetics [11, 12]. Methemoglobinemia is diagnosed by pulse oximetry, patient symptoms, and ABG with CO-oximetry [7]. Oxygen saturation gap between SaO_2_ measured in ABG and SpO_2_ predicts methemoglobinemia [7]. In calculating SaO_2_, all the hemoglobin is assumed normal, whereas SpO_2_ shows the percentage of oxyhemoglobin compared with the total hemoglobin [7]. In this regard, methylene blue and ascorbic acid are used to treat hyperoxia [2]. Methylene blue, which is not used in hemolysis and G6PD deficiency, oxidized the nicotinamide adenine dinucleotide phosphate (NADPH) and reduced to leukomethylene blue, which turns methemoglobin to hemoglobin [7]. Ascorbic acid acts directly as an electron donor and reduces methemoglobin to hemoglobin [2]. Considering the strengths of the current study, in this case acquired methemoglobinemia was accompanied with hemolysis, which was not detected in previous studies and is more likely to be noticed in patients presenting with methemoglobinemia. At first, due to cultural concerns, the patient avoided letting us know about the genital warts, and this could have deviated us from the correct diagnosis; thus, cultural differences should be observed.

## Conclusion

Commonly used drugs may cause acquired methemoglobinemia. Over-the-counter topical anesthetics, including benzocaine and lidocaine, are supposed to induce methemoglobinemia. Self-administration of these drugs is an important issue that may cause life-threatening conditions. Thus, methemoglobinemia should be considered in patients presenting with dyspnea, dizziness, hypoxia, and cyanosis.

## Data Availability

The datasets analyzed in the current study are available from the corresponding author on reasonable request.

## Abbreviations

OTC: Over the counter
ABG: Arterial blood gas
Hb: Hemoglobin
G6PD: Glucose-6-phosphate dehydrogenase
SpO_2_: Saturation of peripheral oxygen
ECG: Electrocardiogram
ESR: Erythrocyte sedimentation rate
CRP: C-reactive protein
HIV: Human immunodeficiency virus
HCV: Hepatitis C virus
HBS: Hepatitis B surface
PT: Prothrombin time
APTT: Activated partial thromboplastin time
INR: International normalized ratio
LDH: Lactate dehydrogenase
pCO_2_: Pressure of CO_2_
pO_2_: Pressure of O_2_
SaO_2_: Arterial oxygen saturation
CNS: Central nervous system
NADPH: Nicotinamide adenine dinucleotide phosphate
